# Comparison of Total Knee Arthroplasty Outcomes Between Renal Transplant and End Stage Renal Disease Patients

**DOI:** 10.5435/JAAOSGlobal-D-21-00288

**Published:** 2022-03-21

**Authors:** Alexandra I. Stavrakis, Alan K. Li, Carlos Uquillas, Christos Photopoulos

**Affiliations:** From the Department of Orthopaedic Surgery, University of California Los Angeles, Los Angeles, CA (Dr. Stavrakis and Mr. Li), and the Department of Orthopaedic Surgery, Cedars-Sinai Kerlan-Jobe Institute, Los Angeles, CA (Dr. Uquillas and Dr. Photopoulos).

## Abstract

Patients with end-stage renal disease (ESRD) have increased risk for periprosthetic joint infection (PJI) due to their predisposition for bacteremia and subsequent implant inoculation secondary to dialysis. PJI risk is also elevated in transplant patients secondary to chronic immunosuppressive therapy. The purpose of this study was to compare medical and surgical complications after primary total knee arthroplasty (TKA) in patients with ESRD or renal transplant (RT).

**Methods:**

This was a retrospective review from the PearlDiver database. International Classification of Diseases and Current Procedural Terminology codes were used to identify patients with ESRD or RT who underwent primary TKA for osteoarthritis from 2015 to 2019. Univariate and multivariable logistic regression analyses were done for medical complications up to 90 days and surgical complications up to 2 years.

**Results:**

Within 90 days of TKA, patients with RT were less likely to develop pneumonia (odds ratio [OR] 0.46, 95% confidence interval [CI] 0.23 to 0.84, *P* = 0.018) and wound dehiscence (OR 0.46, 95% CI 0.21 to 0.90, *P* = 0.015). Patients with RT had a lower risk for PJI at 1 year (OR 0.61, 95% CI 0.36 to 0.99, *P* = 0.017) and at 2 years (OR 0.56, 95% CI 0.34 to 0.88, *P* = 0.017) after primary TKA.

**Discussion:**

Consideration should be given to delaying TKA in patients with ESRD who are RT candidates.

Periprosthetic joint infection (PJI) remains one of the most devastating complications of arthroplasty, oftentimes necessitating multiple surgeries, an extended course of antibiotics, and inferior clinical outcomes. It is also the most common indication for revision total knee arthroplasty (TKA), accounting for more than 25% of these procedures.^1,2^ Revision TKAs not only place a notable financial burden on the United States healthcare system^3^ but also lead to clinically notable reductions in quality of life when compared with primary TKA.^4^ Moreover, when comparing outcomes of revision TKA for PJI versus non-PJI etiologies, PJI revision TKA is associated with worse knee range of motion and patient-reported outcomes scores and even increased mortality.^1,5^ Zmistowski et al^1^ found that revision TKA for PJI had a 10.6% and 25.9% mortality rate at 1 and 5 years, respectively. This was lower than 5-year relative survival rates of the most common malignancies, including prostate cancer, breast cancer, and melanoma.

Several risk factors have been associated with an increased risk of PJI, including obesity, liver disease, diabetes, and renal disease.^6,7^ The relationship between renal disease and PJI is not fully understood, but it has been hypothesized that electrolyte imbalances^8^ and the blunting of the immune system caused by uremia and systemic inflammation are possible mechanisms.^9-11^ Moreover, as the condition progresses to renal failure, the two treatment options available, dialysis and renal transplant (RT), both further increase PJI risk. Dialysis access sites in patients with end-stage renal disease (ESRD) are a potential source of bacteremia and therefore total joint arthroplasty (TJA) implant seeding. RT requires chronic immunosuppressive therapy to prevent transplant rejection, thereby increasing PJI risk. This is particularly problematic because both treatments have been associated with amyloid deposition, osteonecrosis, renal osteodystrophy, and osteoarthritis, all of which increase the need for TJA.^6,12-15^ Thus, a deeper understanding of the interplay between ESRD, RT, and TJA outcomes is necessary to better care for patients who simultaneously have a greater need for TJA and a greater risk for adverse complications after TJA.

Previous studies evaluating PJI risk in patients undergoing these two treatments for renal failure have consisted of small cohorts, lacked longitudinal follow-up, and included total hip arthroplasty and TKA patients together.^15-17^ The purpose of this study was to compare the medical and surgical complications after primary TKA in patients with ESRD or RT.

## Methods

This study was a retrospective review of the MKnee data set of the PearlDiver Patient Record Database (PearlDiver [www.pearldiver.inc]). The MKnee subset is a commercial registry of 1.5 million insured patients who have undergone knee procedures from October 2015 through December 2019. Clinical diagnoses from patient records can be queried using International Classification of Diseases (ICD) and Current Procedural Terminology codes. All data are deidentified and are considered exempt from Institutional Review Board requirements.

ICD-10 and Current Procedural Terminology codes were used to identify patients who had undergone primary TKA. From this group, we only analyzed patients who had undergone surgery for osteoarthritis as identified by the use of ICD-9 and ICD-10 diagnosis codes (Appendix, http://links.lww.com/JG9/A185). Patients without a minimum 2-year follow-up were excluded from the study.

Demographic data for each record included the patient's age (reported as 5-year bins), sex, year of procedure, and comorbidities. Comorbidities were defined when at least one instance of comorbidity was diagnosed under any encounter in the patient record within 1 year before index TKA. The two cohorts, dialysis-dependent patients, herein after referred to as patients with ESRD, and patients with RT, were then queried to identify those who had postoperative complications. Medical complications were collected as prerecorded categories using the PearlDiver analysis package and analyzed up to 90 days after TKA. ICD-10 codes, including those for periprosthetic fracture (PPF), PJI, aseptic loosening, and stiffness, were used to identify surgical complications within 90 days, 1 year, and 2 years from the index procedure (Appendix, http://links.lww.com/JG9/A185).

Data analysis was done using the PearlDiver R analysis package (PearlDiver [www.pearldiver.inc]). Raw/unadjusted univariate analysis of demographic, comorbidity, and complications data was done using χ2 testing and Welch's *t*-test where appropriate. Multivariable logistic regression was done for medical and surgical outcomes of interest while controlling for age, sex, Charlson Comorbidity Index, and all other factors found to be markedly different in the univariate analysis.

## Results

### Patient Demographics

There were 3533 patients with ESRD and 646 patients with RT included in the study. Women made up a larger proportion of the ESRD group (59.81%) versus the RT group (50.31%) (*P* < 0.001). Patients with RT undergoing TKA tended to be younger than patients with ESRD undergoing TKA. A larger percentage of patients in the ESRD group had a Charlson Comorbidity Index ≤3 (50.64% ESRD versus 41.33% RT, *P* < 0.001) (Table 1).

**Table 1:** Baseline Patient Demographics and Charlson Comorbidity Index

Patient Demographics	ESRD (N = 3533)		Renal Transplant (N = 646)		*P*
	N	%	N	%	
Age					
<40	10	0.28	<11	N/A	N/A
40-49	61	1.73	30	4.64	<0.001
50-59	414	11.72	151	23.27	<0.001
60-69	1038	29.38	261	40.40	<0.001
70-79	1856	52.53	190	29.41	<0.001
≥80	154	4.36	<11	N/A	N/A
Sex					
Male	1420	40.19	321	49.69	<0.001
Female	2113	59.81	325	50.31	<0.001
CCI					
≤ 3	1789	50.64	267	41.33	<0.001
4	365	10.33	112	17.34	<0.001
5	381	10.78	90	13.93	0.023
6	271	7.67	59	9.13	0.235
≥ 7	727	20.58	118	18.27	0.197

CCI = Charlson Comorbidity Index, ESRD = end-stage renal disease, N/A = not available

### Baseline Patient Comorbidities

Patients with RT were less likely to have a history of hypertension (79.41% RT versus 86.78% ESRD, *P* < 0.001), coronary artery disease (20.12% RT versus 29.30% ESRD, *P* < 0.001), anemia (9.91% RT versus 15.85% ESRD, *P* < 0.001), acute myocardial infarction (4.02% RT versus 6.99% ESRD, *P* = 0.007), peripheral vascular disease (10.83% RT versus 16.76% ESRD, *P* < 0.001), and cardiac arrhythmias (15.94% RT versus 24.79% ESRD, *P* < 0.001) (Table 2).

**Table 2:** Baseline Patient Comorbidities

Patient Comorbidities	ESRD (N = 3533)		Renal Transplant (N = 646)		*P*
	N	%	N	%	
Cardiovascular					
Hypertension	3066	86.78	513	79.41	<0.001
Coronary artery disease	1035	29.30	130	20.12	<0.001
Anemia	560	15.85	64	9.91	<0.001
Congestive heart failure	234	6.62	51	7.89	0.274
Acute myocardial infarction	247	6.99	26	4.02	0.007
Peripheral vascular disease	592	16.76	70	10.83	<0.001
Cardiac arrhythmias	876	24.79	103	15.94	<0.001
Coagulopathy	235	6.65	43	6.66	1
Pulmonary					
Asthma	312	8.83	41	6.35	0.044
Chronic pulmonary disease	846	23.94	94	14.55	<0.001
Metabolic					
Diabetes	1876	53.10	280	43.34	<0.001
Obesity	1031	29.18	143	22.14	<0.001
Liver disease	252	7.13	65	10.06	0.01
Hypothyroidism	738	20.89	90	13.93	<0.001
Cancer	359	10.16	48	7.43	0.037
Other					
Tobacco use	179	5.07	21	3.25	0.059
Alcohol use	81	2.29	12	1.86	0.586
Drug abuse	140	3.96	17	2.63	0.128
Depression	582	16.47	87	13.47	0.06

ESRD = end-stage renal disease

Patients with RT were less likely to have a history of asthma (6.35% RT versus 8.83% ESRD, *P* = 0.044) and chronic pulmonary disease (14.55% RT versus 23.94% ESRD, *P* < 0.001). Patients with RT were less likely to have diabetes (43.34% RT versus 53.10% ESRD, *P* < 0.001), obesity (22.14% RT versus 29.18% ESRD, *P* < 0.001), hypothyroidism (13.93% RT versus 20.89% ESRD, *P* < 0.001), and cancer (7.43% RT versus 10.16 ESRD, *P* = 0.037) (Table 2).

Liver disease was more common in patients with RT (10.06%) compared with patients with ESRD (7.13%), *P* = 0.01. No other notable differences in baseline comorbidities were observed between patients with RT and ESRD (Table 2).

### Medical Complications

Patients with a history of RT were less likely to develop pneumonia (PNA) within 90 days of TKA (odds ratio [OR] 0.46, 95% confidence interval [CI] 0.23 to 0.84, *P* = 0.018). Patients with RT were also less likely to develop wound dehiscence within 90 days of index TKA (OR 0.46, 95% CI 0.21 to 0.90, *P* = 0.015). No difference was observed between the ESRD and RT groups for risk of deep vein thrombosis (DVT), hematoma, transfusion, urinary tract infection, or sepsis (Table 3).

**Table 3:** Frequency and Adjusted Odds Ratio of Medical Complications at 90 Days

Patient Comorbidities	ESRD (N = 3533)		Renal Transplant (N = 646)		Transplant Odds Ratio (95% Confidence Interval)	*P*
	N	%	N	%		
DVT	40	1.13	11	1.70	0.73 (0.28-1.89)	0.518
Pulmonary embolism	<11	N/A	0	0	N/A	N/A
Pneumonia	129	3.65	11	1.70	0.46 (0.23-0.84)	0.018
Hematoma	21	0.59	<11	N/A	0.84 (0.30-2.32)	0.733
Transfusion	109	3.09	17	2.63	0.89 (0.52-1.52)	0.673
Urinary tract infection	276	7.81	49	7.59	1.08 (0.77-1.50)	0.652
Sepsis	65	1.84	16	25.00	1.40 (0.76-2.44)	0.252
Wound dehiscence	78	2.21	12	1.86	0.46 (0.21-0.90)	0.015

ESRD = end-stage renal disease

### Surgical Complications

Within 90 days of TKA, the RT group was more likely to develop knee stiffness (OR 1.36, 95% CI 1.02 to 1.80, *P* = 0.034). No statistically significant difference was observed in risk of PJI, PPF, or aseptic loosening at 90 days (Table 4). At 1 year, the RT group was less likely to develop a PJI than the ESRD group (OR 0.61, 95% CI 0.36 to 0.99, *P* = 0.017). The RT group was more likely to develop stiffness at 1 year (OR 1.32, 95% CI 1.11 to 1.71, *P* = 0.046). No statistically significant difference was observed for risk of PPF and aseptic loosening between the two groups at 1 year (Table 5). The RT group also had a lower risk of PJI at 2 years (OR 0.56, 95% CI 0.34 to 0.88, *P* = 0.017). No notable difference was observed in risk of PPF, stiffness, or aseptic loosening at 2 years (Table 6).

**Table 4:** Frequency and Adjusted Odds Ratio of Surgical Complications at 90 days

Patient Comorbidities	ESRD (N = 3533)		Renal Transplant (N = 646)		Transplant Odds Ratio (95% Confidence Interval)	*P*
	N	%	N	%		
Periprosthetic joint infection	94	2.66	11	1.70	0.54 (0.27-0.98)	0.057
Periprosthetic fracture	22	0.62	<11	N/A	0.28 (0.02-1.40)	0.223
Stiffness	277	7.84	73	11.3	1.36 (1.02-1.80)	0.034
Aseptic loosening	<11	N/A	0	0	N/A	N/A

ESRD = end-stage renal disease, N/A = not available

**Table 5:** Frequency and Adjusted Odds Ratio of Surgical Complications at 1 Year

Patient Comorbidities	ESRD (N = 3533)		Renal Transplant (N = 646)		Transplant Odds Ratio (95% Confidence Interval)	*P*
	N	%	N	%		
Periprosthetic joint infection	133	3.76	19	2.94	0.61 (0.36-0.99)	0.017
Periprosthetic fracture	26	0.74	<11	N/A	0.46 (0.07-1.58)	0.30
Stiffness	322	9.11	82	12.69	1.32 (1.11-1.71)	0.046
Aseptic loosening	16	0.45	<11	N/A	N/A	N/A

ESRD = end-stage renal disease, N/A = not available

**Table 6:** Frequency and Adjusted Odds Ratio of Surgical Complications at 2 years

Patient Comorbidities	ESRD (N = 3533)		Renal Transplant (N = 646)		Transplant Odds Ratio (95% Confidence Interval)	*P*
	N	%	N	%		
Periprosthetic joint infection	183	5.18	21	3.25	0.56 (0.34-0.88)	0.017
Periprosthetic fracture	32	0.91	<11	N/A	0.39 (0.06-1.31)	0.199
Stiffness	333	9.43	83	12.85	1.28 (0.98-1.67)	0.067
Aseptic loosening	27	0.76	<11	N/A	0.15 (0.01-0.71)	0.062

ESRD = End-stage renal disease, N/A = not available

## Discussion

Because outcomes after RT continue to improve, more patients with ESRD are undergoing this procedure. However, there remains some concern in doing TJA in this patient population because both dialysis and RT have been associated with increased complications and worse outcomes, including increased DVT, PJI, implant loosening, and mortality.^15,16,18,19^ Current literature comparing TJA complications and outcomes between patients receiving dialysis and RT is lacking, and most are limited by small sample sizes.^15-17^ Furthermore, the current available studies that do include larger patient cohorts are limited by short-term outcomes.^12^ In our retrospective cohort study, we included a much larger patient cohort with the 2-year follow-up.

Compared with patients with ESRD, patients with RT had a nearly onefold decrease in risk of PJI at both 1 and 2 years after TKA. A similar difference was observed at 90 days after TKA, although statistical significance was not reached (*P* = 0.057). In 2015, Cavanaugh et al^12^ demonstrated decreased rates of surgical site infection after TJA in patients with RT compared against dialysis patients. In a more recent single center study, Inoue et al^20^ also reported a decreased risk of surgical site infection or PJI after TJA in patients with RT compared with dialysis patients. Although these two studies grouped both hip and knee arthroplasty patients together, the results of our study, which evaluated TKA patients only, are consistent with their findings. It is also important to note that these previous studies only reported postoperative infection rates during the index hospitalization or within 90 days after surgery. Thus, our current study builds on these observations by focusing on TKA and by demonstrating that the differences in risk persist up to 2 years after index arthroplasty. Our findings support the suggestions made by previous studies to consider RT before TJA in patients with severe kidney disease.^12,16,17,20^

Interestingly, patients with RT were found to have increased knee stiffness at 90 days and 1 year after TKA. This is a cause for concern because patients with arthrofibrosis have poor functional outcomes and increased knee pain,^21,22^ both of which are major determinants of patient satisfaction with TKA.^23^ Arthrofibrosis is characterized by excessive proliferation of scar tissue.^24^ Although the precise mechanism of pathogenesis is unclear, it has been posited that derangements in type-I collagen deposition during scar formation or fibrinolysis during scar remodeling play a role in joint contracture.^25,26^ Following this line of thought, one possible explanation for increased knee stiffness observed in patients with RT in our study is that the use of glucocorticoids—one of the most common agents in combination immunosuppressive therapy after solid organ transplant—has been shown to diminish fibrinolysis.^27^ The resultant inadequate tissue remodeling could contribute to stiffness in patients with RT. Although arthrofibrosis is associated with worse postoperative outcomes, the morbidity associated with PJI is graver.^1,2^ The previously mentioned decrease in risk of PJI arguably outweighs the higher rates of stiffness in patients with RT. As such, consideration should be given to delaying TKA in patients with ESRD who are RT candidates.

Although our study found that rates of medical complications were similar between patients with ESRD and RT, the latter cohort did demonstrate markedly lower rates of PNA within 90 days of TKA. This is important because studies have shown that chronic kidney disease is a risk factor for community-acquired and nosocomial PNA,^28^ and patients with kidney disease experience more severe bouts with this lower respiratory tract infection.^29^ Moreover, PNA is one of the most common nonsurgical complications after surgery,^30^ including after TJA surgery.^31^ Postoperative PNA prolongs length of stay after surgery, increases medical costs, and is associated with increased morbidity and mortality.^30,32,33^ This is yet another reason why clinicians may consider RT before TKA in transplant candidates to avoid placing patients, who already have an increased predisposition for acquiring PNA, at even greater risks.

There are some limitations to our current study that should be considered. First, as a retrospective database study, there is an inherent possibility for inaccuracies in data entry and coding. Second, we were unable to identify the microorganism responsible for PJI, which may be an important factor to consider. Finally, information regarding implant design and fixation technique was unavailable.

Despite these limitations, it is our understanding that this study is the first to investigate longitudinal differences in rates of medical and surgical complications between these two patient cohorts, specifically in patients with TKA . Our study demonstrates that compared with patients with ESRD, patients with RT are at a decreased risk for PJI after TKA. This finding is consistent with results from smaller previous studies with short-term outcomes.^12,16,17,20^ In addition, our results also agree with the recommendations made by the second International Consensus Meeting on orthopaedic infections to conduct TJA after solid organ transplant to reduce rates of PJI.^34^ Although not all patients with ESRD are candidates for RT, those who are should be made aware of the differences in complication and infection risks to avoid adverse outcomes and further improve patient outcomes with TKA.

## References

[1] Zmistowski B Karam JA Durinka JB Casper DS Parvizi J: Periprosthetic joint infection increases the risk of one-year mortality. J Bone Joint Surg Am 2013;95:2177-2184. 24352771 10.2106/JBJS.L.00789

[2] Bozic KJ Kurtz SM Lau E : The epidemiology of revision total knee arthroplasty in the United States. Clin Orthop 2010;468:45-51. 19554385 10.1007/s11999-009-0945-0 PMC2795838

[3] Kurtz SM Lau E Watson H Schmier JK Parvizi J: Economic burden of periprosthetic joint infection in the United States. J Arthroplasty 2012;27(8 suppl):61-65, e1. 22554729 10.1016/j.arth.2012.02.022

[4] Greidanus NV Peterson RC Masri BA Garbuz DS: Quality of life outcomes in revision versus primary total knee arthroplasty. J Arthroplasty 2011;26:615-620. 20541360 10.1016/j.arth.2010.04.026

[5] Wang C-J Hsieh M-C Huang T-W Wang J-W Chen H-S Liu C-Y: Clinical outcome and patient satisfaction in aseptic and septic revision total knee arthroplasty. The Knee 2004;11:45-49. 14967328 10.1016/S0968-0160(02)00094-7

[6] Kim C-W Kim H-J Lee C-R Wang L Rhee SJ: Effect of chronic kidney disease on outcomes of total joint arthroplasty: A meta-analysis. Knee Surg Relat Res 2020;32:12. 32660587 10.1186/s43019-020-0029-8 PMC7219208

[7] Zhu Y Zhang F Chen W Liu S Zhang Q Zhang Y: Risk factors for periprosthetic joint infection after total joint arthroplasty: A systematic review and meta-analysis. J Hosp Infect 2015;89:82-89. 25575769 10.1016/j.jhin.2014.10.008

[8] Schatz V Neubert P Schröder A : Elementary immunology: Na+ as a regulator of immunity. Pediatr Nephrol Berl Ger 2017;32:201-210. 10.1007/s00467-016-3349-x PMC5203836 26921211

[9] Lamarche C Iliuta I-A Kitzler T: Infectious disease risk in dialysis patients: A transdisciplinary approach. Can J Kidney Health Dis 2019;6:2054358119839080. 31065378 10.1177/2054358119839080 PMC6488776

[10] Chonchol M: Neutrophil dysfunction and infection risk in end-stage renal disease. Semin Dial 2006;19:291-296. 16893406 10.1111/j.1525-139X.2006.00175.x

[11] Betjes MGH Litjens NHR: Chronic kidney disease and premature ageing of the adaptive immune response. Curr Urol Rep 2015;16:471. 25404185 10.1007/s11934-014-0471-9

[12] Cavanaugh PK Chen AF Rasouli MR Post ZD Orozco FR Ong AC: Complications and mortality in chronic renal failure patients undergoing total joint arthroplasty: A comparison between dialysis and renal transplant patients. J Arthroplasty 2016;31:465-472. 26454568 10.1016/j.arth.2015.09.003

[13] Li J Li M Peng B Luo R Chen Q Huang X: Comparison of total joint arthroplasty outcomes between renal transplant patients and dialysis patients—a meta-analysis and systematic review. J Orthop Surg 2020;15:590. 10.1186/s13018-020-02117-3 PMC7724818 33298121

[14] Erkocak OF Yoo JY Restrepo C Maltenfort MG Parvizi J: Incidence of infection and inhospital mortality in patients with chronic renal failure after total joint arthroplasty. J Arthroplasty 2016;31:2437-2441. 27341974 10.1016/j.arth.2016.04.031

[15] García-Ramiro S Cofán F Esteban PL : Total hip arthroplasty in hemodialysis and renal transplant patients. HIP Int 2008;18:51-57. 18645975 10.1177/112070000801800110

[16] Shrader MW Schall D Parvizi J McCarthy JT Lewallen DG: Total hip arthroplasty in patients with renal failure: A comparison between transplant and dialysis patients. J Arthroplasty 2006;21:324-329. 16627138 10.1016/j.arth.2005.07.008

[17] Lieberman JR Fuchs MD Haas SB : Hip arthroplasty in patients with chronic renal failure. J Arthroplasty 1995;10:191-195. 7798100 10.1016/s0883-5403(05)80126-3

[18] Sakalkale DP Hozack WJ Rothman RH: Total hip arthroplasty in patients on long-term renal dialysis. J Arthroplasty 1999;14:571-575. 10475556 10.1016/s0883-5403(99)90079-7

[19] Sunday JM Guille JT Torg JS: Complications of joint arthroplasty in patients with end-stage renal disease on hemodialysis. Clin Orthop 2002;397:350-355. 10.1097/00003086-200204000-00040 11953627

[20] Inoue D Yazdi H Goswami K Tan TL Parvizi J: Comparison of postoperative complications and survivorship of total hip and knee arthroplasty in dialysis and renal transplantation patients. J Arthroplasty 2020;35:971-975. 31870581 10.1016/j.arth.2019.10.038

[21] Anania A Abdel MP Lee Y Lyman S González Della Valle A: The natural history of a newly developed flexion contracture following primary total knee arthroplasty. Int Orthop 2013;37:1917-1923. 23835560 10.1007/s00264-013-1993-3 PMC3779550

[22] Morrey ME Abdel MP Riester SM : Molecular landscape of arthrofibrosis: Microarray and bioinformatic analysis of the temporal expression of 380 genes during contracture genesis. Gene 2017;610:15-23. 28167142 10.1016/j.gene.2017.01.025

[23] Hamilton DF Lane JV Gaston P : What determines patient satisfaction with surgery? A prospective cohort study of 4709 patients following total joint replacement. BMJ Open 2013;3:e002525. 10.1136/bmjopen-2012-002525 PMC3641464 23575998

[24] Kalson NS Borthwick LA Mann DA : International consensus on the definition and classification of fibrosis of the knee joint. Bone Joint J 2016;98-B:1479-1488. 27803223 10.1302/0301-620X.98B10.37957

[25] Abdel MP Morrey ME Barlow JD : Myofibroblast cells are preferentially expressed early in a rabbit model of joint contracture. J Orthop Res 2012;30:713-719. 22057979 10.1002/jor.21588

[26] Klement MR Penrose CT Bala A Wellman SS Bolognesi MP Seyler TM: How do previous solid organ transplant recipients fare after primary total knee arthroplasty? J Arthroplasty 2016;31:609-615, e1. 26639984 10.1016/j.arth.2015.10.007

[27] Sáez-Giménez B Berastegui C Loor K : Deep vein thrombosis and pulmonary embolism after solid organ transplantation: An unresolved problem. Transpl Rev 2015;29:85-92. 10.1016/j.trre.2014.12.005 25573688

[28] McDonald HI Thomas SL Millett ERC Nitsch D: CKD and the risk of acute, community-acquired infections among older people with diabetes mellitus: A retrospective cohort study using electronic health records. Am J Kidney Dis 2015;66:60-68. 25641062 10.1053/j.ajkd.2014.11.027 PMC4510204

[29] James MT Quan H Tonelli M : CKD and risk of hospitalization and death with pneumonia. Am J Kidney Dis 2009;54:24-32. 19447535 10.1053/j.ajkd.2009.04.005

[30] Kazaure HS Martin M Yoon JK Wren SM: Long-term results of a postoperative pneumonia prevention program for the inpatient surgical ward. JAMA Surg 2014;149:914. 25054486 10.1001/jamasurg.2014.1216

[31] Bozic KJ Grosso LM Lin Z : Variation in hospital-level risk-standardized complication rates following elective primary total hip and knee arthroplasty. J Bone Joint Surg 2014;96:640-647. 24740660 10.2106/JBJS.L.01639

[32] Warren DK Shukla SJ Olsen MA : Outcome and attributable cost of ventilator-associated pneumonia among intensive care unit patients in a suburban medical center. Crit Care Med 2003;31:1312-1317. 12771596 10.1097/01.CCM.0000063087.93157.06

[33] Rello J Ollendorf DA Oster G : Epidemiology and outcomes of ventilator-associated pneumonia in a large US database. Chest 2002;122:2115-2121. 12475855 10.1378/chest.122.6.2115

[34] Azboy I Bedair H Demirtas A : General assembly, prevention, risk mitigation, general factors: Proceedings of international consensus on orthopedic infections. J Arthroplasty 2019;34:S55-S59. 30348580 10.1016/j.arth.2018.09.054

